# New Paradigm in the Role of Regulatory T Cells During Pregnancy

**DOI:** 10.3389/fimmu.2019.00573

**Published:** 2019-03-26

**Authors:** Sayaka Tsuda, Akitoshi Nakashima, Tomoko Shima, Shigeru Saito

**Affiliations:** Department of Obstetrics and Gynecology, University of Toyama, Toyama, Japan

**Keywords:** miscarriage, preeclampsia, pregnancy, regulatory T cells, seminal plasma

## Abstract

Semi-allogenic fetuses are not rejected by the maternal immune system because feto-maternal tolerance induced by CD4^+^CD25^+^FoxP3^+^ regulatory T (Treg) cells is established during pregnancy. Paternal antigen-specific Treg cells accumulate during pregnancy, and seminal plasma priming plays an important role in expanding paternal antigen-specific Treg cells in mouse models. Although paternal-antigen specific Treg cells have not been identified in humans, recent studies suggest that antigen-specific Treg cells exist and expand at the feto-maternal interface in humans. Studies have also revealed that reduction of decidual functional Treg cells occurs during miscarriage with normal fetal chromosomal content, whereas insufficient clonal expansion of decidual Treg cells is observed in preeclampsia. In this review, we will discuss the recent advances in the investigation of mechanisms underlying Treg cell-dependent maintenance of feto-maternal tolerance.

## Introduction

Feto-maternal tolerance protects the fetal tissues from rejection and leads to a successful pregnancy (1–7). After implantation of the blastocyst in the uterine endometrium, trophoblasts start to invade the endometrial tissue, and uterine spiral artery. Maternal lymphocytes such as CD4^+^ T cells, CD8^+^ T cells, and CD16^−^CD56^bright^ natural killer (NK) cells express activation markers on their surfaces, suggesting that maternal lymphocytes recognize trophoblasts or fetuses (8). Interaction with maternal immune regulation and trophoblast-derived tolerogenic molecules induces a tolerogenic environment at the feto-maternal interface. Considering the maternal immune system, regulatory T cells (Treg cells) play an essential role in the maintenance of allogenic pregnancy (9–12). CD4^+^CD25^+^Foxp3^+^ regulatory T (Treg) cells regulate the T cell response. Treg cells are necessary to sustain tissue homeostasis and establish immune tolerance (13), and are also related to tumor growth and organ transplantation tolerance (14). Previous studies in mouse models have demonstrated that paternal antigen-specific Treg cells are expanded systemically and locally during pregnancy (15–17). Seminal plasma primes the induction of paternal antigen-specific Treg cells (17, 18). Treg cells also increase systemically and locally during human pregnancies (12, 19), whereas paternal antigen-specific Treg cells have not been identified in humans. Recent studies show that target-specific, clonally expanded Treg cells are expanded at the feto-maternal interface in human pregnancies (20). In the first part of this review, we discuss mechanisms by which Treg cells induce feto-maternal tolerance and highlight antigen-specific Treg cells by introducing recent important findings. Following that, we will attempt to analyze the relationship between maldistribution and dysfunction of Treg cells and implantation failure, recurrent pregnancy loss, and preeclampsia in humans.

### Maternal Immune Cells at the Feto-Maternal Interface

**Figure 1:**
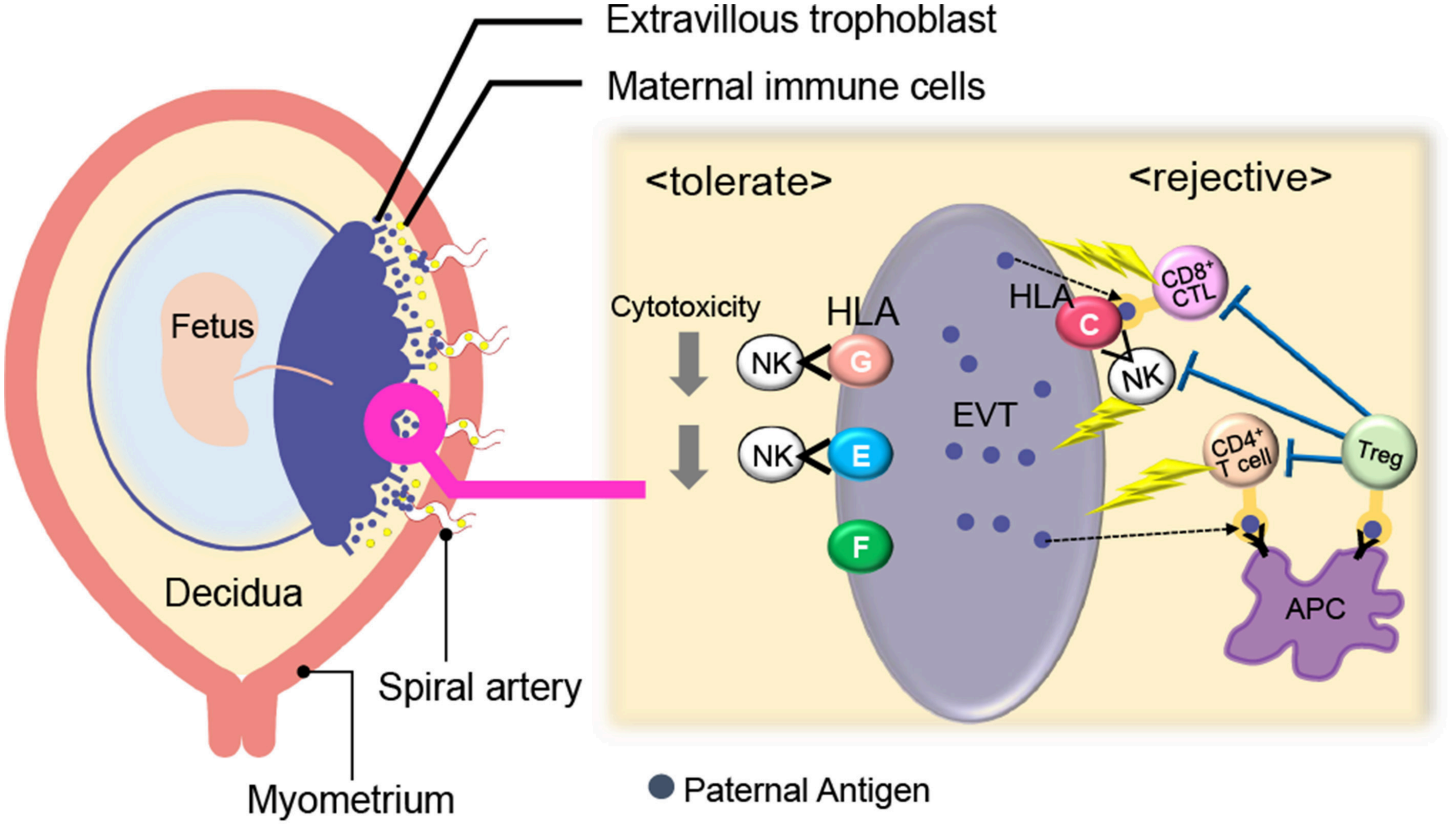
Immunological balance at the feto-maternal interface during early pregnancy. EVTs did not express polymorphic HLA-A, B whereas HLA-C and non-polymorphic HLA-E, G, and F were expressed. Maternal CD8^+^ T cells and NK cells can directly recognize paternal HLA-C and CD4^+^ T cells can indirectly recognize it. HLA- E and G protect EVTs from NK-cell mediated cytotoxicity. Treg cells can recognize fetal antigens via maternal antigen presenting cells (APCs) and induce tolerance in an antigen-specific manner. EVT, Extravillous trophoblast; NK, natural killer cell; Treg; regulatory T cell; APC, antigen-presenting cell.

Maternal immune cells in the reproductive tissues first come into contact with paternal antigens when seminal fluid is ejaculated into the vagina during intercourse. Seminal fluid is composed of seminal plasma and sperm. Maternal immune cells recognize paternal antigens which are contained in the seminal plasma. Sperm reach the fallopian tube and fertilize the oocyte present there. After fertilization, the blastocyst migrates to the uterus while undergoing cell cleavage and finally attaches to the decidua. During the implantation period, the blastocyst adheres to and starts invading the uterine endometrium. In human pregnancy, the cells of the trophoblast differentiate into villous and extravillous trophoblasts (EVTs), forming the placenta. EVTs invade the decidua and myometrium. Subsequent to implantation, EVTs further penetrate the maternal spiral artery and finally replace the vascular lumen (21, 22). The feto-maternal interface is thereby formed, and EVTs and maternal immune cells contact each other (23). EVTs escape from maternal immune cells by controlling the major histocompatibility complex (MHC) and expressing immune suppressive molecules. The maternal immune system also dynamically changes to induce tolerance against fetal tissues (Figure 1).

Villous trophoblasts lack the surface expression of MHC class I and class II. EVTs do not express polymorphic HLA-A, B, whereas they express HLA-C and non-polymorphic HLA-E, G, and F (24–29). Maternal CD8^+^ T cells and NK cells can directly recognize paternal HLA-C, and CD4^+^ T cells can indirectly recognize it. On the other hand, HLA- E and G protect EVTs from NK-cell mediated cytotoxicity (30, 31). HLA-G positive EVTs regulate T cell activation through the induction of tolerogenic dendritic cells (DCs) (32) and directly cause the expansion of Treg cells (33). Furthermore, trophoblasts suppress maternal immune cells via the expression of indoleamine 2,3-dioxygenase (IDO) (34, 35), the secretion of inhibitory cytokines, such as IL-10 and TGF-β (36), and the expression of programmed death ligand (PD-L I) (37).

Considering maternal immune cells in the decidua, Treg cells and CD56^bright^CD16^−^uterine NK (uNK) cells play an important role in the maintenance of feto-maternal tolerance (3, 4, 38–41) (Figure 1). Treg cells, which are discussed in detail at a later part, can recognize fetal antigens via maternal antigen presenting cells (APCs) and induce tolerance in an antigen-specific manner. Compared with ordinary peripheral blood (pb) NK cells that have high cytotoxicity, uNK cells (CD56^bright^CD16^−^ NK cells) produce many cytokines and their cytotoxic activities are low (42). Rather, uNK cells play an important role in uterine angiogenesis and spiral artery remodeling (23, 38, 43–46). The expression patterns of cell surface NK receptors in uNK cells differ from those of pbNK cells. For example, killer immunoglobulin-like receptor (KIR) and natural killer group 2 (NKG2) receptors are expressed at high levels on uNK cells (47). When the NKG2A receptor recognizes HLA-E on EVTs, an inhibitory signal suppresses the cytotoxicity of uNK cells (26). When KIR2DL on uNK cells interacts with HLA-G, the uNK cell activity is suppressed (30, 33).

CD8^+^ cytotoxic T cells can recognize the fetal antigen directly via HLA-C on EVTs and indirectly via maternal APCs (Figure 1). A previous report showed that fetal antigen-specific CD8^+^ cytotoxic T cells (CTLs) are detected in maternal peripheral blood during human pregnancies (48, 49). Viral antigen-specific decidual CTLs that can cross-react against allo-antigens are also reported (50, 51). CTLs in the decidua have distinct phenotypes and functions compared with those in peripheral blood. T-cell immunoglobulin mucin-3 (Tim-3) and programmed cell death-1 (PD-1) are negative immune regulatory molecules. The expression of Tim-3^+^PD-1^+^CD8^+^ T cells was higher in the human decidua than in peripheral blood. EVTs promote enrichment of Tim-3^+^PD-1^+^CD8^+^ T cells in an HLA-C dependent manner, suggesting that decidual CD8^+^ T cells would not attack trophoblasts. Furthermore, maternal Tim-3^+^PD-1^+^CD8^+^ T cells recognize PD-L I expressed on EVTs, resulting in trophoblast antigen-specific tolerance (52). Highly differentiated resident memory CD8^+^ T cells are observed in the decidua. This subset shows a lower expression of perforin and granzyme B (53). These reports suggest that antigen-specific CTLs exist at the feto-maternal interface, but their cytotoxic activity is controlled by the placental tissue (53).

### How Do Paternal Antigen-Specific Treg Cells Function in Allogenic Pregnancy?

Previous studies suggest that Treg cells play an essential role in the induction of paternal antigen-specific tolerance in allogenic pregnancy in mice. Paternal MHC-specific tolerance during allogenic pregnancy was demonstrated by Tafuri et al. where a paternal MHC-bearing tumor graft was not rejected during pregnancy with the conceptus MHC being identical to the tumor graft, but was rejected in the postpartum period (54).

Aluvihare et al. (11) demonstrated that Treg cells are necessary for allogenic pregnancy in mice, but not necessary for syngeneic pregnancy. When T cell-depleted BALB/C nu/nu female mice were mated with C57BL/6 male mice (allogenic pregnancy) after transfer of total lymphocytes, pregnancies were normally maintained. On the other hand, mating after transfer of Treg cell-depleted lymphocytes resulted in fetal loss, suggesting that Treg cells are essential for the maintenance of allogenic pregnancies (11). Adoptive transfer of CD4^+^CD25^+^ Treg cells from mice with an allogenic pregnancy prevented fetal rejection in an abortion-prone mouse model during allogenic pregnancy, if the transfer was conducted before day 4.5 of gestation (9). Furthermore, depletion of Treg cells using anti-CD25 monoclonal antibodies induced implantation failure and abortion in allogenic pregnancies, but did not induce any pregnancy complications during the late stages of pregnancy (10). These findings suggest that Treg cells induce allo-antigen-specific tolerance and are necessary from implantation through early pregnancy periods in mice.

**Figure 2:**
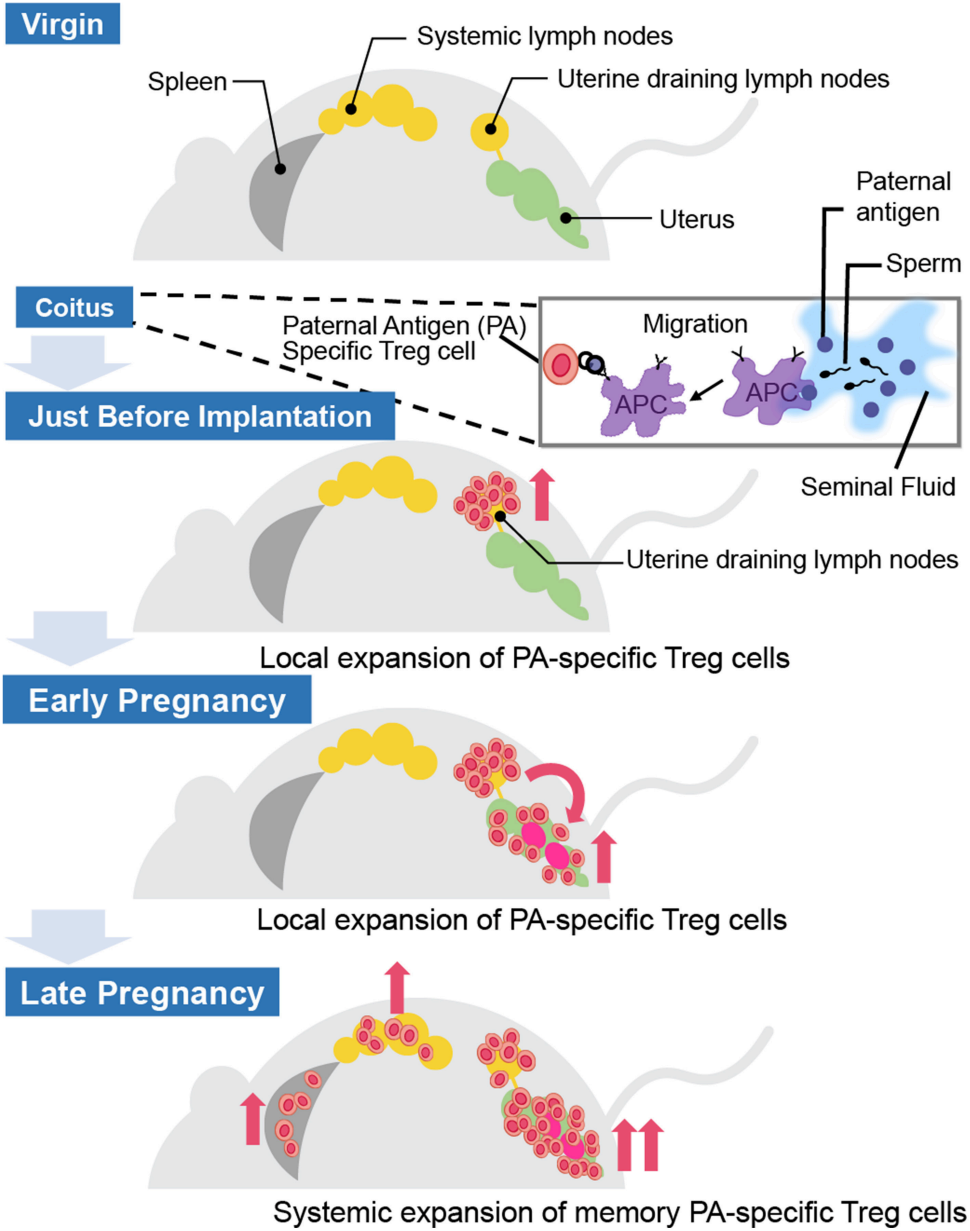
Distribution of paternal antigen-specific Treg cells in mice. When a female mouse is mated with an allogenic male mouse, paternal antigen-specific Treg cells increase in the uterine-draining lymph nodes one day before implantation due to seminal plasma priming. After implantation, the paternal antigen-specific Treg cells population in the uterus increases day by day during the course of the pregnancy, but not in the peripheral lymph nodes and the spleen. APC, antigen-presenting cell.

Where and how do fetal antigen-specific Treg cells expand during pregnancy? Previous studies have demonstrated the existence of fetal antigen-specific Treg cells and their distribution in mouse models (Table 1). Kahn and Baltimore showed that the H-Y-specific suppressive capability of Treg cells in splenocytes escalated more during pregnancy than before pregnancy (15). Rowe et al. demonstrated that Treg cells specific for the 2W1S antigen, which is derived from the mouse MHC-Eα chain, expanded in the systemic lymph nodes during the 1st pregnancy and rapidly re-accumulated during the 2nd pregnancy (16). Furthermore, Shima et al. reported the local distribution of paternal antigen-specific Treg cells during the implantation period and after pregnancy (Figure 2). DBA/2 mice have the Mls 1a super antigen, which is recognized by Vβ6 of the T cell receptor β chain. When BALB/C female mice were mated with DBA/2 male mice, CD4+CD25+Vβ6+ Treg cells, which can be regarded as Mls 1a-specific Treg cells, increased in the uterine-draining lymph nodes one day before implantation. This phenomenon was not observed when BALB/C female mice were mated with seminal vesicle-excised DBA/2 male mice. The local fetal antigen-specific Treg cells might be expanded at the draining lymph nodes by seminal plasma-priming and migrate to the uterus after pregnancy. After implantation, the Vβ6+ Treg cell population in the uterus increased day by day during pregnancy, but that in peripheral lymph nodes and spleen did not (17). Therefore, accumulation of paternal antigen-specific Treg cells is regulated in an organ-specific manner.

**Table 1:** Paternal antigen-specific Treg cells in mouse models.

**Target Antigen**	**PA-specific Treg cells**	**Method**	**Existence of PA-antigen specific Treg cells**	**References**
H-Y antigen derived peptide (Dby)	MHC classII I-A^b^ H-Y (Dby_608−622_)restricted Treg cells	MLR	Systemic	(15)
I-A^b^ restricted 2W1S_52−68_ peptide	MHC classII I-Ab 2W1S_52−68_ restricted Treg cells	Direct detection by tetramer	Syetemic	(16)
Mls 1a superantigen	CD4+CD25+Vβ6+ Treg cells (Vβ6 of the T cell receptor β chain recognize Mls 1a superantigen	Direct detection by anti-Vβ6 antibody	Uterine-draining lymph nodes, Uterus	(17)

*PA-specific Treg cells, Paternal antigen-specific Treg cells; MLR, Mixed lymphocyte reaction*.

### Paternal Antigen-Specific Treg Cells in Human Pregnancies

During pregnancy in humans, the systemic and local expansion of the Treg cell pool is observed from the 1st trimester and reaches a maximum in the 2nd trimester (12, 19). Although fetal antigen-specific regulatory T cells and their systemic and local expansion were observed in a mouse model, direct detection of fetal antigen-specific Treg cells is difficult in humans due to heterogenic MHC expression and limited knowledge concerning the physiological target peptide of Treg cells (55). However, the existence of fetal antigen-specific Treg cells in human pregnancies was indirectly suggested in some reports.

**Figure 3:**
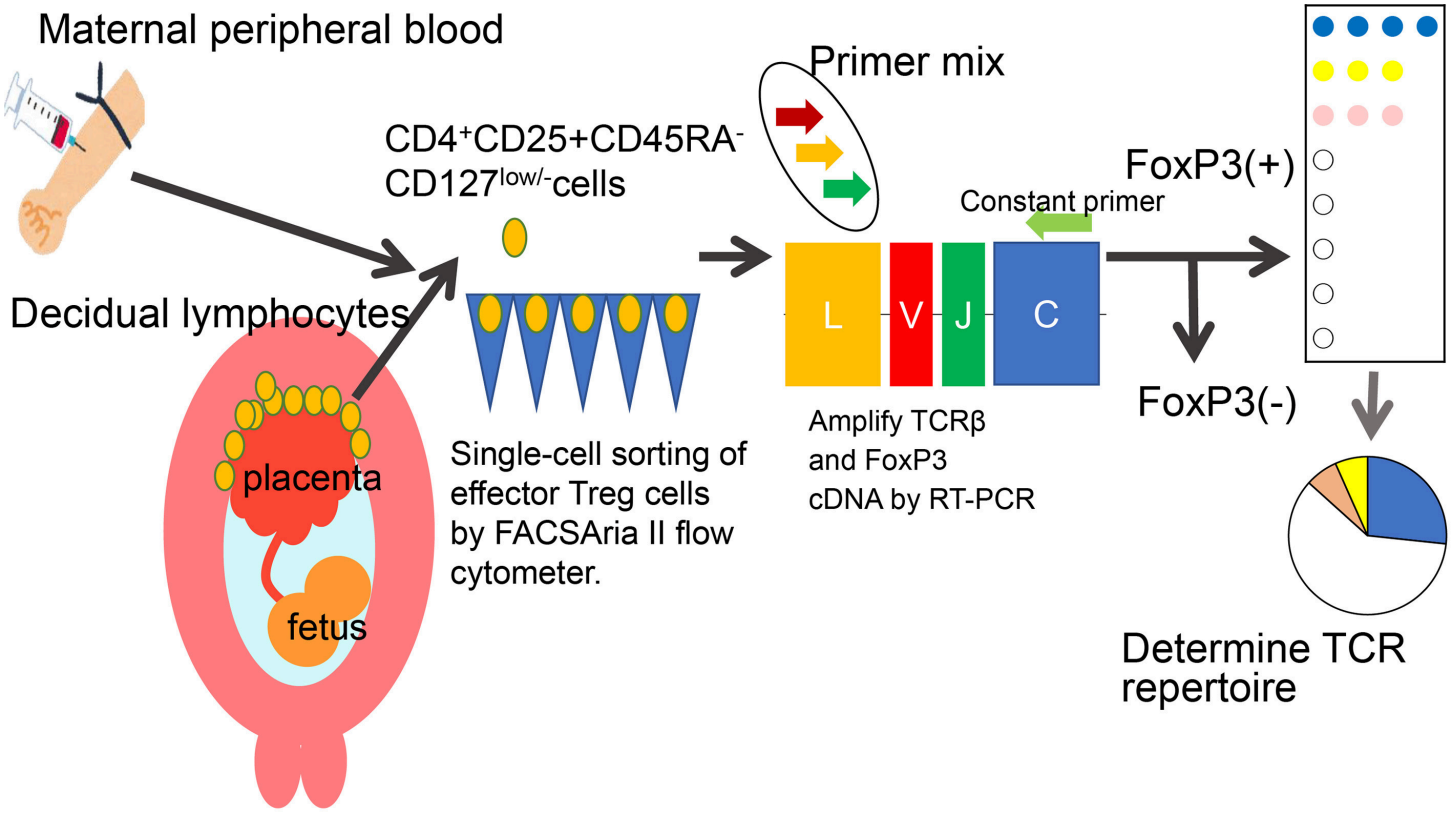
Single-cell based TCR repertoire analysis method. To study the clonality of effector Treg cells, a single-cell based T cell receptor (TCR) repertoire analysis method was used. Paired samples of maternal peripheral blood mononuclear cells and decidual lymphocytes were obtained. CD4+CD25+CD45RA-CD127low/- effector Treg cells were single-cell sorted. The cDNAs of complementarity determining lesion 3 (CDR3) in TCRβ chain and FoxP3 were amplified by RT-PCR. The nucleotides and amino acid sequences of CDR3 were analyzed.

**Figure 4:**
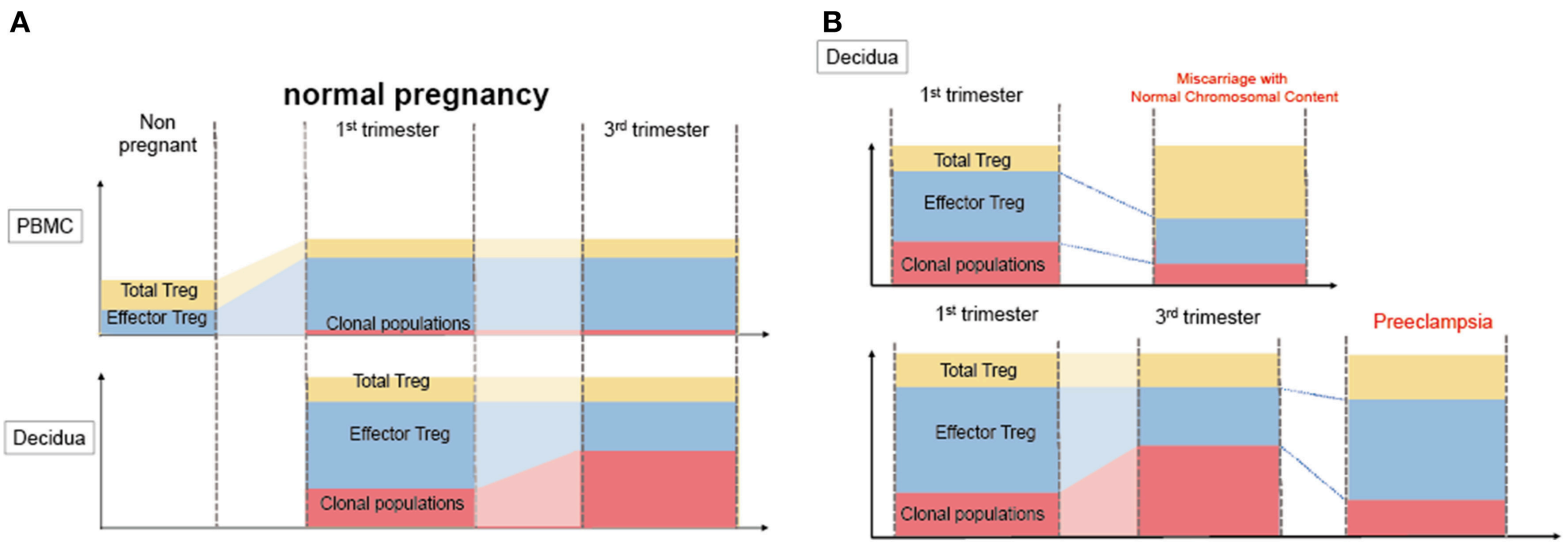
Distribution of clonally expanded effector Treg cells in humans. **(A)** Frequencies of total Treg cells, effector Treg cells and clonal populations of effector Treg cells in normal pregnancy. The systemic and local expansion of the Treg cells and effector Treg cells pool is observed during pregnancies in humans. Clonally expanded effector Treg cells increase in decidua, but not in peripheral blood. Clonal populations of effector Treg cells more increase in 3rd trimester than 1st trimester. **(B)** Clonally expanded decidual effector Treg cells in miscarriage and preeclampsia. In decidua, effector Treg cells pool decreased in miscarriage with normal chromosomal content than 1st trimester normal pregnancy, whereas the frequency of clonal populations of effector Treg cells does not significantly decrease. On the other hand, clonal populations of decidual effector Treg cells decreased preeclampsia than 3rd trimester normal pregnancies.

Decidual Treg cells, but not peripheral blood Treg cells, showed higher suppression toward self-fetal cord blood than 3rd party cord blood, suggesting that fetal antigen-specific Treg cells might exist at the feto-maternal interface during human pregnancy (56). Among human Treg cell subsets, CD4^+^CD45RA^−^FoxP3^high^ comprises effector Treg cells which are memory type T cells with a high suppressive capability, and CD4^+^CD45RA^+^FoxP3^low^ comprises naïve Treg cells with a relatively lower suppressive capability (57, 58). Effector Treg cells are the most dominant among Treg cells in both peripheral blood and decidua in the late gestation stage of human pregnancies (59). To demonstrate if expansion of the effector Treg cell pool is a reflection of clonal expansion of antigen-specific Treg cells, we conducted single-cell-based T cell receptor (TCR) repertoire analysis of CD4^+^CD25^+^CD45RA^−^CD127^low^ effector Treg cells in human pregnancies (Figure 3). Our study was the first to reveal that clonally expanded effector Treg cells were observed only in the decidua, but not in the peripheral blood (Figure 4A). Clonally expanded effector Treg cells were higher in the 3rd trimester than in the 1st trimester (Figure 4A). On the other hand, the common clonotypic effector Treg cells between the decidua and peripheral blood were rarely observed (20). Therefore, decidual effector Treg cells might recognize some antigens expressed at the feto-maternal interface and proliferate upon antigen stimulation. However, effector Treg cells in the peripheral blood expand nonspecifically. Interestingly, the same clonotypic decidual effector Treg cells repeatedly appeared in previous and subsequent pregnancies in three cases: two ended with paired normal term deliveries and the third ended with paired miscarriages (20). TCRβ varies, with over 2 × 10^7^ patterns estimated in young humans (60), thus these same clonotypic Treg cells might be repeatedly recruited by the same antigens at the feto-maternal interface rather than by accidental coincidence. Furthermore, clonal populations of decidual effector Treg cells were lower in the 3rd trimester in preeclampsia cases than in normal pregnancies (Figure 4B). However, while the effector Treg cell pool was reduced, clonal populations were not reduced in 1st trimester miscarriage cases (20) (Figure 4B). Taken together, these data indicate that fetal antigen-specific Treg cells might be recruited and expand in a fetal antigen-specific manner at the feto-maternal interface, and polyclonally expand in systemic circulation during human pregnancy. Clonally expanded decidual Treg cells might be important in the maintenance of feto-maternal tolerance, especially in the 3rd trimester.

### Which Peptides Are Recognized by Treg Cells at the Feto-Maternal Interface?

Paternal MHC and minor antigen-derived peptide-specific Treg cells were identified in mouse models as previously described (15–17). A recent study showed that non-inherited maternal antigen (NIMA)-specific Treg cells enforce tolerance in a mouse model (61). NIMAs are peptides derived from polymorphic genes such as MHC and are expressed in the mother but not in offspring. Microchimerism, developed during pregnancy and breast feeding, enables transfer of maternal cells to the offspring. Microchimeric maternal cells persist for a long time and tolerance for NIMAs persists during this time. Kinder et al. demonstrated that the paternal MHC-derived antigen was identical to the NIMA, and the rapid expansion of NIMA-specific Treg cells contributed to successful pregnancy (61). NIMA-matched organ transplantation presents a lower risk of rejection than NIMA-mismatched transplantation in humans (62–65). Thus, theoretically, NIMA-specific tolerance might be induced during human pregnancy.

In humans, a polymorphic HLA-C mismatch pregnancy indicates T cell activation and Treg cell expansion (66). In an oocyte donation (OD) pregnancy, in which the fetus is a total allograft, the match level of HLA-A, B, C, DR, and DQ between the mother and offspring was higher, and fewer pregnancy complications were observed (67). Trophoblasts lack surface expression of HLA-class II molecules, but contain them intracellularly. Considering microchimerism between the mother and fetus, peptides derived from HLA-class II can act as epitopes presented by maternal APCs. Trophoblasts contain intracellular HLA-class II and release HLA-DR molecules upon stimulation by IFN-γ (68). Cell surface expression of HLA-DR in syncytiotrophoblasts and the presence of HLA-DR in syncytiotrophoblast-derived extracellular vesicles were observed in preeclampsia (69). Seminal plasma also contains soluble HLA molecules (70–72). Additionally, human EVTs express minor histocompatibility antigens, such as HY, HA, and ACC (73). HY antigen-specific CD8^+^ T cells were observed during human pregnancy (48). Even these minor histocompatibility antigens mediate graft-vs.-host disease after organ transplantation (73); however, fetal tissues are not rejected. Taken together, allogenic-HLA-derived, and minor histocompatibility antigen-derived peptides presented by maternal APCs might be recognized by CD4^+^ conventional T cells and Treg cells. The main target antigens of decidual CD4^+^ conventional T cells, CTLs, and Treg cells are yet unclear. Further investigation is required to reveal the antigen-specificity of each T cell type and their regulation at the feto- maternal interface.

### What Is the Origin of Treg Cells at the Feto-Maternal Interface?

There are two types of Treg cells in terms of origin: naturally occurring Treg (nTreg) cells, which originate in the thymus, and inducible Treg (iTreg) cells, which arise from conventional CD4^+^ T cells in peripheral tissues (74). Conserved noncoding sequence 1 (CNS1) is a FoxP3 enhancer and is necessary for developing iTreg cells. Interestingly, only placental mammals have CNS-1, while marsupials and monotremes do not. CNS-1 knockout female mice with allogenic pregnancies showed increased fetal resorption (75). Thus, iTreg cells might be necessary to maintain allogenic pregnancy in placental mammals. On the other hand, nTreg cells are the dominant population (~95%) among decidual Treg cells in the 1st trimester of human pregnancy, and the proportion of nTreg cells is similar between normal pregnancy and miscarriage (76). However, decidual iTreg cells significantly decreased in preeclampsia (77). To maintain human pregnancy, nTreg cells might be important in early stage pregnancy, and iTreg cells might also be important in late-stage gestation. Further study is necessary to confirm this possibility.

### Pregnancy Complications and Treg Cells in Humans

Maldistribution and functional impairment of Treg cells were reported in implantation failure, miscarriage, and preeclampsia in humans. Contrarily, Treg cells are necessary in the implantation period and early gestation, but not in late gestation in mice (10).

Multiple factors, including Treg cell impairment, are thought to be related to implantation failure in humans. Treg cell transcription factor FoxP3 mRNA expression in the uterine endometrium is decreased in primary unexplained infertility (78, 79). A decrease in Treg cells in the peripheral blood in the late follicular phase predicts failure of artificial insemination by the donor (AID) sperm (80). These findings support the hypothesis that maldistribution of Treg cells impairs implantation in humans. Additionally, exposure to seminal plasma raises the success rate of IVF-ET pregnancy (81). Further investigation can validate the evidence that priming with the seminal plasma results in Treg cell-mediated tolerance and can rescue implantation failure.

**Figure 5:**
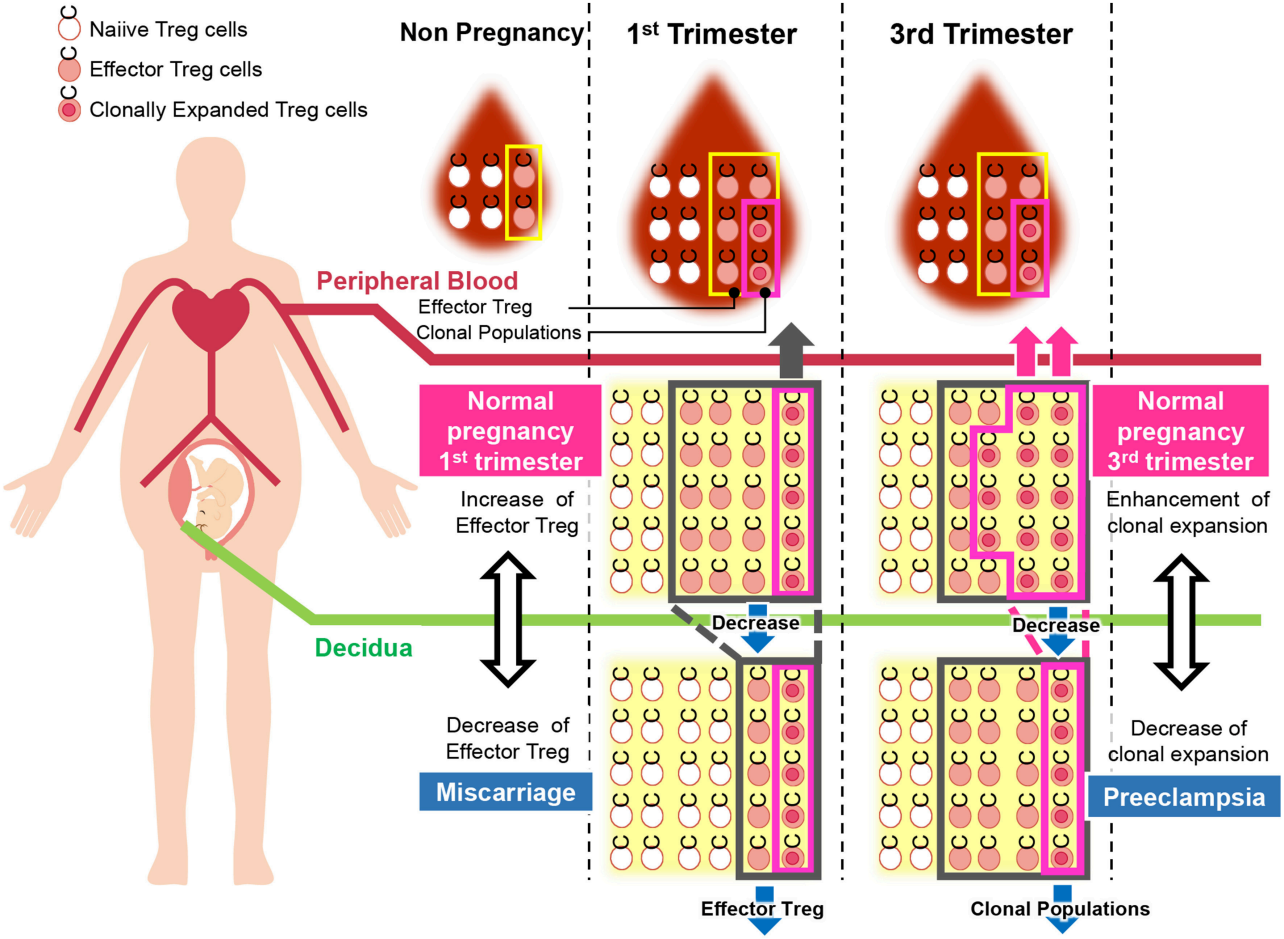
Pathological change in Treg cells during pregnancy in humans. During pregnancy, Treg cell pools both in the peripheral blood and decidua expand. Clonally expanded effector Treg cells are observed only in the decidua, but not in the peripheral blood. Clonally expanded effector Treg cells are higher in the 3rd trimester than in the 1st trimester. In miscarriage cases with normal chromosomal fetal content, the number of decidual effector Treg cells decreases. On the other hand, clonal populations of decidual effector Treg cells decrease in cases of preeclampsia.

Disturbance of Treg cell-mediated tolerance might be one of the etiologies of miscarriage. Previous studies reported that Treg cells were decreased in the peripheral blood and decidua in miscarriage cases (12, 82–84). Impaired suppressive capability of Treg cells in recurrent miscarriage cases has also been observed (85–88). Effector Treg cells and nTreg cells were decreased in the case of miscarriage with a normal karyotyped fetus compared to that in the 1st trimester of a normal pregnancy or in the case of miscarriage with an abnormal karyotyped fetus (76, 89). On the other hand, the clonally expanded population of Treg cells showed no significant difference between these groups (20) (Figure 5). These findings suggest that the number of nTreg cells and effector Treg cells is more important than antigen-specific Treg cell recruitment during the 1st trimester. The total Treg cell volume that regulates excessive inflammation might be important for the maintenance of the early gestation phase of pregnancy. A previous report demonstrated that high dose immunoglobulin therapy improved the live birth rate for refractory recurrent miscarriage cases with four or more consecutive miscarriages (90). Other studies also showed the effectiveness of anti-TNF-alfa inhibitor therapy (91). These medications have the potential to suppress immune activity in an antigen-nonspecific manner; therefore, these therapies might benefit patients with recurrent miscarriage with a reduced decidual effector Treg cell pool. In humans, peripheral blood Treg cells and decidual Treg cells form clonotypically different populations, and the migration of Treg cells from systemic circulation to the decidua has not yet been shown (20). Thus, these findings might explain why immunization therapy using white blood cells from the patient's partner is not effective for treating recurrent miscarriage (92).

Preeclampsia, which is defined as hypertension concomitant with proteinuria or placental dysfunction occurring in mid to late gestation, is a major cause of maternal and fetal morbidity and mortality. Chronic inflammation due to activation of neutrophils and NK cells, elevation of pro-inflammatory cytokines, and dysfunction of Treg cells is also thought to contribute to the pathogenesis of preeclampsia (93). Epidemiological findings provide the hypothesis that failure in maintaining paternal antigen-specific tolerance is related to the development of preeclampsia. First pregnancy, pregnancy following a partner change, and a pregnancy interval of more than ten years raise the risk of preeclampsia (94–96). Long-term condom usage and AID pregnancy also elevate the risk of preeclampsia, suggesting insufficient paternal antigen-specific tolerance mediated by seminal plasma priming (70, 97, 98). OD pregnancy, in which the fetus is completely allogenic and no priming effect has occurred, is associated with a high risk of preeclampsia (6, 98). Basic research on Treg cells supports this hypothesis.

Previous reports show that Treg cell pools decrease in the peripheral blood and decidua in preeclampsia (77, 99–108). Some reports demonstrate functionally impaired Treg cells in preeclampsia, where Treg cell apoptosis can be easily induced (109). Other reports showed that effector Treg cells decreased in the peripheral blood (108). Hsu et al. demonstrated that the function of decidual APCs was impaired in preeclampsia, resulting in fewer peripherally induced Treg cells (iTreg cells) than in normal pregnancies (77). Elevation of soluble endoglin (sEND), which is a co-receptor of TGFβ, results in the capture of circulating TGFβ, resulting in a systemic decrease of the Treg cell pool. It might also disturb the conversion of conventional Treg cells to iTreg cells (6).

So far, it has not been clarified whether a decreased total volume of Treg cells or decreased paternal antigen-specific Treg cells are related to the pathogenesis of preeclampsia. Our study reported for the first time that clonal expansion of decidual Treg cells was impaired in preeclampsia, suggesting that paternal antigen-specific tolerance might be insufficient. The frequencies of clonal populations of decidual effector Treg cells were 20.9% (15.4–28.1%) in the 3rd trimester during normal pregnancy and 9.3% (4.4–14.5%) in pregnancies with preeclampsia (Figure 5). Both early onset and late onset preeclampsia showed the same tendency (20). Our result is compatible with epidemiological evidence that inadequate paternal antigen-specific tolerance raises the risk of preeclampsia. Paternal antigen-specific Treg cells are more important in the late gestation period of pregnancy than in early gestation (Figure 3). Decidual iTreg cells and clonal populations of decidual effector Treg cells decreased in preeclampsia (20, 77). The main population of decidual effector Treg cells during early pregnancy was that of nTreg (76), and the clonal population of decidual effector Treg cells did not decrease. These findings suggest that clonally expanded Treg cells might be iTreg cells. This point requires clarification in the future.

In terms of the clinical applications of these findings, oocyte donation after HLA matching with maternal or paternal HLA might reduce the risk of preeclampsia. Encouraging seminal plasma exposure might play a protective role for high risk patients.

## Conclusion

Treg cell-mediated feto-maternal tolerance is important in the maintenance of allogenic pregnancy. Paternal antigen-specific Treg cells are expanded systemically and locally during mouse pregnancy. Seminal plasma priming induces paternal antigen-specific Treg cells. Although paternal antigen-specific Treg cells have not been identified in humans, clonal expansion of decidual effector Treg cells implies that antigen-specific tolerance by Treg cells might be induced during human pregnancy. A reduced amount of decidual Treg cells might be related to the pathogenesis of miscarriage, and the failure of decidual Treg cell clonal expansion might be related to the pathogenesis of preeclampsia in humans.

## Author Contributions

SS, ST, TS, and AN conception and design, drafting manuscript and revision of the manuscript for important intellectual content.

### Conflict of Interest Statement

The authors declare that the research was conducted in the absence of any commercial or financial relationships that could be construed as a potential conflict of interest.
